# Efficiency of an mHealth App and Chest-Wearable Remote Exercise Monitoring Intervention in Patients With Type 2 Diabetes: A Prospective, Multicenter Randomized Controlled Trial

**DOI:** 10.2196/23338

**Published:** 2021-02-09

**Authors:** Jing Li, Dong Wei, Shuyi Liu, Mingxia Li, Xi Chen, Li Chen, Yuelei Wu, Wen Zhou, Lingyun Ouyang, Cuixia Tan, Hongdao Meng, Nanwei Tong

**Affiliations:** 1 Department of Endocrinology and Metabolism West China Hospital of Sichuan University Chengdu China; 2 Department of Endocrinology Chengdu Second People’s Hospital Chengdu China; 3 Department of Endocrinology Chengdu No 1 People’s Hospital Chengdu China; 4 Department of Endocrinology Chengdu Branch in Tibet of Huaxi Hospital affiliated with Sichuan University Chengdu China; 5 Recovery Plus Clinic Chengdu China; 6 School of Aging Studies College of Behavioral and Community Sciences University of South Florida Tampa, FL United States

**Keywords:** type 2 diabetes, fitness app, heart rate band, exercise monitoring, randomized controlled trial, mobile phone

## Abstract

**Background:**

Exercise has been recommended as a cornerstone for diabetes management. Supervised exercise is more efficient than unsupervised exercise but is less convenient and accessible.

**Objective:**

We aimed to determine the efficiency of exercise using a fitness app and heart rate band to remotely monitor patients with type 2 diabetes in comparison with that of traditional exercise.

**Methods:**

Patients with type 2 diabetes without severe complications or comorbidities were recruited to participate in this multicenter randomized controlled trial and were allocated to either the intervention or control group (1:1 ratio). Participants in both groups were asked to engage in moderate to vigorous physical activity for at least 150 minutes per week; each participant was prescribed individualized exercises. Participants in the intervention group were asked to follow exercise videos on the app and to wear a chest band; heart rate, exercise duration, and exercise intensity were recorded by the app. Participants in the control group self-reported exercise intensity and duration. Cardiopulmonary endurance, body composition, blood glucose level, and insulin level were assessed before and after a 3-month exercise program.

**Results:**

Of the 101 participants who were enrolled, the majority of them (85/101, 84.2%) completed the study. Both groups had similar baseline characteristics, with the exception that participants in the intervention group were slightly younger and less likely to have hypertension. Self-reported exercise duration was longer than app-recorded exercise duration (control: mean 214 minutes/week; intervention: mean 193 minutes/week); in addition, a higher proportion of participants in the control group (29/41, 71%) than in the intervention group (18/44, 41%) met the 150-minute target for moderate to vigorous physical activity. However, compared with the control group, the intervention group had a larger increase in cardiopulmonary endurance (mean difference –2.0 bpm [beats per minute] vs 1.0 bpm; *P*=.02) and a larger decrease in body fat percentage (mean difference –1.8% vs –0.8%; *P*=.01). There was no difference in hemoglobin A1c level reduction between the two groups, yet more participants in the intervention group stopped taking their antidiabetic drugs or had their dosages lowered by an endocrinologist, compared with those in the control group. There were no serious adverse events in either group.

**Conclusions:**

This was the first randomized controlled trial in China, to our knowledge, to test the efficiency of exercise using a fitness app and heart rate band to remotely monitor prescribed exercise in patients with type 2 diabetes. The findings of our study suggest that exercise programs may be more efficient if participants are remotely monitored with an app and heart rate band than if participants are not monitored.

**Trial Registration:**

Chinese Clinical Trial Register ChiCTR1800015963; http://www.chictr.org.cn/showprojen.aspx?proj=27080

## Introduction

Cardiovascular disease and microvascular complications related to type 2 diabetes are significant causes of premature mortality and morbidity, resulting in a heavy economic burden [1,2]. Lack of physical activity is a risk factor for the development of type 2 diabetes [3], but exercise is effective in both preventing and managing type 2 diabetes [4]; exercise improves blood glucose, reduces cardiovascular risk factors, and improves cardiorespiratory fitness [5]. Therefore, exercise has been recommended as a cornerstone of diabetes management [6,7].

Many factors, including exercise frequency, intensity, duration, type, and volume, have an influence on the efficiency of exercise [8]. Individuals are likely to benefit more from regular exercise that is longer in duration, of greater intensity, or both [9]. Consequently, accurate monitoring and quantification of exercise intensity, duration, and adherence affect exercise efficiency. Supervised exercise, such that the features of exercise and its adherence are monitored by trained staff, is typically performed in specialized facilities. Although unsupervised exercise is more convenient, it may be less efficient. One study [10] found that, compared with participants engaged in unsupervised exercise, those who engaged in supervised aerobic and resistance exercise demonstrated greater improvements in hemoglobin A_1c_ (HbA_1c_) level, blood pressure, BMI, waist circumference, and other metabolic parameters. The American Diabetes Association recommends supervised exercise, when feasible, for patients with type 2 diabetes [8].

However, the majority of patients with diabetes do not have access to supervised exercise, because it requires trained staff, typically requires specific facilities, and is more expensive than unsupervised exercise. The widespread use of fitness apps and wearable technology in recent years may facilitate the objective monitoring of physical activity. To our knowledge, no studies have examined the efficiency of exercise that is monitored using a fitness app with wearable technology in patients with type 2 diabetes in China. We hypothesized that exercise supervised through remote monitoring using a fitness app and heart rate band would be more efficient than traditional unsupervised exercise in improving cardiopulmonary endurance, body composition, and blood glucose level control in type 2 diabetes patients.

## Methods

### Study Design

We conducted a prospective, multicenter randomized controlled trial (RCT); the trial was registered with the Chinese Clinical Trial Registry (ChiCTR1800015963). We recruited participants from four tertiary care hospitals—West China Hospital, Chengdu Second People’s Hospital, Chengdu First People’s Hospital, and Chengdu Branch in Tibet of Huaxi Hospital Affiliated with Sichuan University—in Chengdu, China, through advertising or from endocrinology clinics from September to October 2018. After telephone screening, eligible participants were invited to an orientation session, in which detailed information about the study was provided to potential participants, and informed consent forms were signed by individuals who agreed to participate. Participants aged 18 to 64 years old were included if they met all of the following criteria:

1. Their type 2 diabetes diagnosis was confirmed with an oral glucose tolerance test after recruitment into the study.
2. They had been diagnosed with diabetes 10 years or less prior to the study.
3. They had access to a smartphone that was capable of running the app used in the study.

We used American Diabetes Association criteria for the diagnosis [6], and we chose a limit of 10 years or less since their diabetes diagnosis in order to exclude individuals with advanced diabetes. In addition, individuals with any of the following conditions were excluded:

1. Fasting plasma glucose level greater than 16.7 mmol/L.
2. Recurring hypoglycemia.
3. A history of acute diabetic complications, including diabetic ketoacidosis, hyperosmolar hyperglycemia, and lactic acidosis.
4. A history of severe chronic micro- or macrovascular complications.
5. Diseases that may be exacerbated by exercise or influence exercise efficiency, such as uncontrolled hypertension, hyperthyroidism, osteoarthritis, or hypokalemia.
6. Another medical condition that was judged by the researchers to preclude participation.

The study was approved by the Research Ethics Board of the West China Hospital of Sichuan University.

### Randomization

After initial screening and signing informed consent forms, participants were randomly assigned to either the intervention group or the control group. Allocation was performed using four sets of pregenerated randomization schedules that corresponded to the four participating hospital outpatient clinics, with varying block sizes of 2, 4, and 6.

### Procedure

Questionnaires were completed by each participant, and exercise testing was performed on every participant before the start of the exercise programs. The questionnaire was designed to collect information about exercise habits, diet, and lifestyle. Exercise tests that were assessed included resting heart rate measurement, step testing, and muscular endurance. All participants were prescribed a target of 150 minutes per week of moderate to vigorous physical activity based on American College of Sports Medicine guidelines [9] and the information provided by questionnaire responses and from exercise tests. Participants were informed that they would likely see greater improvements if they were able to achieve a target of 300 minutes of moderate to vigorous physical activity or above per week. Both aerobic and resistance exercise, with at least 10 minutes of aerobic exercise each session, were recommended to every participant.

Participants in the intervention group downloaded the R Plus Health app (Recovery Plus Inc), which connected wirelessly to a chest-worn heart rate band (Recovery Plus Inc) to measure exercise frequency, intensity, time, volume, and progression. Although prescribed exercise programs were explained to each participant verbally by the researchers at the start of the study, participants in the intervention group were also sent exercise programs through the app. Individualized exercises were demonstrated with videos on the app. Participants in the intervention group were asked to follow the exercise videos on the app and wear the heart rate band when exercising to determine whether they had reached their target heart rate. The target heart rate (*HR*_target_), estimated using the heart rate reserve method, was calculated as follows:

*HR*_target_ = (*HR*_max_ – *HR*_rest_) × *intensity*% (40% to 90%) + *HR*_rest_

The maximum heart rate (*HR*_max_) was estimated as follows:

*HR*_max_ = 220 – *age*

and resting heart rate (*HR*_rest_) was measured during initial exercise testing. The app notified the user during exercise as to whether they needed to adjust their workload to achieve the target heart rate, automatically calculated the cumulative time of exercise that reached the target heart rate, and showed the remaining amount of exercise required for that week to meet the target. The user interface of the R Plus Health app is shown in Multimedia Appendix 1, Figures S1-S4.

Participants in the control group self-reported exercise intensity, which they determined using a modified Borg Scale of Perceived Exertion. A diary was given to the participants in the control group to record the exercise duration and intensity, and the information in the diary was collected during monthly follow-ups.

Participants in both groups were followed up by telephone every month. Blood glucose levels were recorded, and endocrinologists reviewed and adjusted medication dosages, if necessary. Participants were followed up for 3 months.

### Outcomes

The primary outcomes were body fat percentage and cardiorespiratory endurance. Secondary outcomes were blood glucose level, insulin level, homeostasis model assessment of insulin resistance (HOMA-IR), muscle strength, and cholesterol level. Primary and secondary outcome measures were assessed before and after the exercise programs. Cardiorespiratory endurance was evaluated using a YMCA 3-minute step test. Muscle strength was assessed using the Baseline hydraulic grip handheld dynamometer, model 12-0240 (Fabrication Enterprises, Inc). Body fat percentage was measured using the Lunar iDXA dual-energy x-ray absorptiometer (GE Healthcare). All the tests were conducted before and after the intervention. Both cardiorespiratory and muscle strength assessments were performed by trained researchers at a study hospital site. Safety events and drug adjustments were recorded for both groups.

### Statistical Analysis

Statistical analyses were performed using Stata, version 15.1 (StataCorp LLC). Either the chi-square test or the Fisher exact test was used for categorical variables, and *t* tests were used for continuous variables. *P* values of .05 or less were considered statistically significant.

## Results

**Figure 1:**
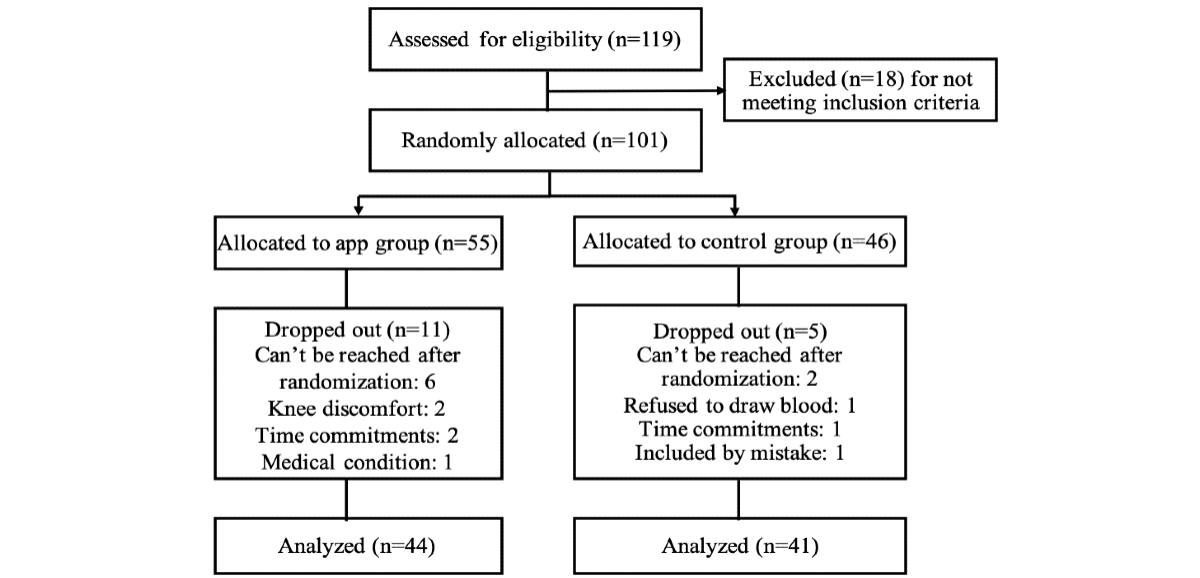
Study flow diagram.

A total of 119 patients were assessed for eligibility, of which 101 individuals met the inclusion criteria and were randomized to either the intervention group (55/101, 54.5%) or the control group (46/101, 45.5%); 85 participants out of 101 (84.2%) completed the study: 44 (52%) from the intervention group and 41 (48%) from the control group. Of the 16 participants out of 101 (15.8%) who did not complete the study, 11 (69%) from the intervention group and 5 (31%) from the control group dropped out of the study (see Figure 1).

Demographic characteristics of the study population are presented in Table 1; the mean age of the participants was 48.2 years (SD 10.4), 76.2% (77/101) were male, and 23.8% (24/101) were female. The mean HbA_1c_ was 7.3% (SD 1.8), the mean BMI was 25.2 kg/m^2^ (SD 3.6), and the mean duration since diabetes diagnosis was 46.9 months (SD 41.5). A total of 24.8% (25/101) of the participants had a history of hypertension and 14.9% (15/101) reported having a sedentary lifestyle. With the exception of age and history of hypertension, both groups had similar baseline characteristics. Participants in the intervention group were significantly younger and were less likely to have a history of hypertension.

**Table 1:** Baseline individual characteristics by treatment group.

Characteristic	Total sample (N=101)	Control group (n=46)	Intervention group (n=55)	*P* value
Number of participants, n (%)	101 (100)	46 (45.5)	55 (54.5)	N/A^a^
Age (years), mean (SD)	48.2 (10.4)	51.7 (8.6)	45.3 (11.0)	.001
Female, n (%)	24 (23.8)	12 (26)	12 (22)	.65
Duration since diagnosis (months), mean (SD)	46.9 (41.5)	49.7 (40.6)	44.7 (42.5)	.55
Smoker, n (%)	38 (37.6)	21 (46)	17 (31)	.15
Use alcohol, n (%)	19 (18.8)	11 (24)	8 (15)	.31
Sedentary, n (%)	15 (14.9)	7 (15)	8 (15)	>.99
Family history of diabetes, n (%)	40 (39.6)	18 (39)	22 (40)	>.99
Stressed, n (%)	3 (3.0)	2 (4)	1 (2)	.59
Hypertension, n (%)	25 (24.8)	16 (35)	9 (16)	.04
Weight (kg), mean (SD)	70.5 (13.8)	68.9 (12.9)	71.9 (14.4)	.26
BMI (kg/m^2^), mean (SD)	25.2 (3.6)	25.0 (3.1)	25.4 (4.0)	.58
Overweight or obese, n (%)	75 (74.3)	34 (74)	41 (75)	>.99
Waist-to-hip ratio, mean (SD)	0.6 (0.1)	0.6 (0.1)	0.5 (0.1)	.21
Body fat (%), mean (SD)	31.3 (7.7)	31.6 (8.1)	31.0 (7.4)	.69
Hemoglobin A_1c_ (%), mean (SD)	7.3 (1.8)	7.5 (1.8)	7.2 (1.8)	.43
Resting heart rate (bpm^b^), mean (SD)	78.8 (8.9)	79.6 (9.1)	78.0 (8.7)	.37
Step test (bpm), mean (SD)	113.9 (15.6)	113.0 (16.0)	114.7 (15.4)	.60

^a^N/A: not applicable; the *P* value was not calculated for this characteristic.

^b^bpm: beats per minute.

Among participants who completed the study, the self-reported duration of exercise in the control group (214 minutes/week) was longer than the app-recorded duration of exercise in the intervention group (193 minutes/week). Similarly, a higher proportion of participants in the control group (29/41, 71%) than in the intervention group (18/44, 41%) met the 150-minute target for moderate to vigorous physical activity; however, compared with the control group, the intervention group had a significantly larger change in cardiorespiratory endurance (mean difference –2.0 bpm [beats per minute] vs 1.0 bpm; *P*=.02) and a significantly larger decrease in body fat percentage (–1.8% vs –0.8%; *P*=.01) as shown in Table 2. Finally, the intervention group had a greater decrease in BMI (–0.60 kg/m^2^ vs –0.32 kg/m^2^); however, the difference was not statistically significant (*P*=.09).

There was no difference in HbA_1c_ level reduction between the two groups (–0.55% and –0.70% in the intervention and control groups, respectively) after exercise intervention. However, 4 participants in the intervention group stopped taking antidiabetic drugs, and 9 participants had their dosages lowered because of well-controlled blood glucose levels, whereas in the control group, 1 participant stopped taking antidiabetic drugs, 2 participants had their dosages lowered, and 5 participants had their dosages increased. There were no significant differences in muscle strength, HOMA-IR, blood cholesterol level, and waist-to-height ratio between the two groups.

There were no severe adverse events in either group. Hypoglycemia occurred 4 times in the intervention group and 8 times in the control group, and muscle pain occurred 8 times in the intervention group and 8 times in the control group.

**Table 2:** Effect of exercise in type 2 diabetes patients in intervention and control groups.

Exercise measure	Intervention group (n=44)	Control group (n=41)	*P* value
BMI (kg/m^2^), median (IQR)	–0.60 (–1.07 to 0.16)	–0.32 (–0.74 to 0.01)	.09
Hemoglobin A_1c_ (%), median (IQR)	–0.55 (–1.53 to –0.07)	–0.70 (–1.40 to 0.40)	.46
Body fat (%), median (IQR)	–1.80 (–2.95 to –0.10)	–0.80 (–1.73 to 0.65)	.01
HOMA-IR^a^, median (IQR)	0.07 (–0.51 to 1.06)	–0.17 (–1.48 to 1.29)	.23
HOMA-β^b^, median (IQR)	1.44 (–20.70 to 21.18)	3.64 (–19.02 to 42.47)	.52
Resting heart rate (bpm^c^), median (IQR)	2.00 (–3.00 to 10.50)	4.00 (–2.00 to 10.00)	.52
Step test (bpm), median (IQR)	–2.00 (–9.50 to 3.00)	1.00 (–3.00 to 10.00)	.02
Muscle strength (repetitions), mean (SD)	–0.90 (4.60)	–0.78 (4.16)	.91

^a^HOMR-IR: homeostasis model assessment for insulin resistance.

^b^HOMA-β: homeostasis model assessment for β-cell function.

^c^bpm: beats per minute.

## Discussion

We found that the majority of participants were able to complete their prescribed exercise program in this study. Although participants in the intervention group demonstrated lower adherence (ie, participated for a shorter duration) than those in the control group, their improvement of cardiorespiratory endurance and decrease in body fat percentage were greater than those of the control group, and more participants in the intervention group stopped taking their antidiabetic drugs or had their dosage of antidiabetic drugs lowered. No severe adverse events were found in either group. These findings suggest that exercise monitored remotely using a fitness app and heart rate band was feasible and efficient among Chinese adults with type 2 diabetes.

There have been many studies showing that exercise interventions improve blood glucose control and other factors, such as weight, body fat mass, cardiorespiratory endurance, and glycemic stability [5,11]; these factors are highly related to long-term cardiovascular diseases and mortality [12,13] in individuals with diabetes. Most studies [5] utilized structured exercise undertaken in specialized facilities with the supervision of trained staff. In such conditions, participants were motivated to adhere to the exercise program by those providing the training, and results cannot be generalized to nonresearch settings where the majority of exercise is unsupervised. Several studies [14,15] have shown that when direct supervision was removed during the maintenance portion of a program, adherence dropped and glycemic control decreased. Studies that compared the efficiency of structured exercise with unstructured exercise found that supervised structured training was more efficient in decreasing HbA_1c_ levels [16]. In our study, exercise performed by the intervention group was monitored using an app and heart rate band. Real-time feedback from the app notified users when to adjust exercise intensity and duration and encouraged exercise adherence. Our results demonstrate that exercise monitored with a fitness app and heart rate band was more efficient than unsupervised exercise in improving cardiorespiratory endurance and decreasing body fat percentage in patients with type 2 diabetes. Because supervised exercise is not accessible for most participants, exercise with remote monitoring using a fitness app and heart rate band can be used as a surrogate.

Mobile app–based interventions for diabetes are widely supported by evidence with high certainty [17]; however, the distribution and efficiency of apps vary by functional design [18,19]. These studies [18,19] recorded exercise duration and intensity by participant self-reporting or objectively using accelerometers. In our study, target heart rate was used to monitor exercise intensity and track exercise duration, which allowed objective assessment of exercise duration and intensity.

Although participants in the intervention group did not have a significantly lower BMI than those in the control group, their decrease in body fat percentage was greater. Previous studies reported a reduction in body fat percentage for their control group that was similar to that of our control group, but a much lower reduction for their intervention group than that of our intervention group [20]. Body fat may be more important than BMI in predicting risk for type 2 diabetes, especially in participants with a BMI value in the normal range, and fat mass is an important factor that contributes to insulin resistance, metabolic syndrome, and other cardiovascular risk parameters [21,22]. Therefore, remotely monitored exercise using a fitness app may be a useful method to improve body composition and avoid metabolic consequences.

Our study found similar HbA_1c_ level reductions to those found by another study [23] that tested the efficiency of aerobic and resistance exercise supervised by trained staff in specialized facilities in patients with type 2 diabetes. It should be noted that we allowed the adjustment of antidiabetic drugs in the participants, which may have resulted in the effect of exercise on HbA_1c_ reduction being underestimated. This suggests that remotely monitored exercise and traditional supervised exercise were similarly efficient in terms of reducing blood glucose levels. Because of the convenience and low cost of monitoring with an app and heart rate band, this option may be a better choice for most patients with type 2 diabetes. Although the intervention group did not demonstrate greater HbA_1c_ reductions than those of the control group, more participants had their antidiabetic medication dosage reduced or stopped, which can lead to more patient engagement and lower out-of-pocket costs.

Although there are studies [5,24] showing that exercise improved muscle strength, HOMA-IR, blood cholesterol level, and waist-to-height ratio, our study did not demonstrate that participants in the intervention group had greater improvements in metabolic parameters than those of the control group. Most of these studies had a duration of 16 weeks and had a control group of sedentary participants. The reason that our study did not demonstrate relative improvements in metabolic parameters may be explained by the small sample size, the short duration, and use of an active control group.

In our study, participants in the control group reported longer exercise durations and better adherence to prescribed exercise programs. Many studies [25,26] have already shown that there are discrepancies between self-reported and objective measures of exercise duration and intensity—individuals are prone to overestimate the duration and intensity of exercise in self-reports; therefore, wearable technology can be used to guarantee accurate assessment of these parameters.

Several limitations should be considered when interpreting our study’s findings. First, exercise duration and intensity in the control group were self-reported and, thus, may not be reliable. Second, there were slight differences in the mean age and percentage of participants with hypertension between the groups. Participants in the intervention group were slightly younger, and there was a lower percentage with hypertension, possibly because those who were older and had higher cardiovascular risk were more likely to decline to participate. Chance, due to the small sample size in this multicenter RCT, may also have contributed to baseline differences that were observed. Studies have shown that older age is associated with a decrease in exercise capacity [27]. Because participants in the control group were older, it is possible that they may have had decreased exercise capacity, which may have resulted in overestimation of exercise efficiency in the control group. However, participants in the control group reported longer exercise durations and higher adherence to the exercise program, and the age difference between the two groups was minimal. In addition, exercise is as efficient in older patients with type 2 diabetes as it is in those who are younger [24]. Therefore, the age difference between groups may not have an impact on the findings of this study.

To the best of our knowledge, this was the first RCT in China that tested the efficiency of exercise monitored remotely using an app and heart rate band in patients with type 2 diabetes. We found that, despite shorter exercise durations and lower adherence to exercise in the intervention group than those in the control group, participants in the intervention group demonstrated greater improvements in cardiorespiratory endurance and greater reductions in body fat percentage. Both groups had clinically meaningful reductions in HbA_1c_, and more participants in the intervention group stopped requiring hypoglycemic drugs or had their hypoglycemic drug dosage lowered. These findings suggest that exercise monitored remotely with an app and heart rate band is more efficient than unsupervised exercise in type 2 diabetes patients. Because of the convenience and low cost of the app and wearable technology, it may be used as a surrogate for traditional supervised exercise.

## Appendix

Multimedia Appendix 1 Screenshots of the study app's user interface.

## Abbreviations

bpm: beats per minute
HbA_1c_: hemoglobin A_1c_
HOMA-IR: homeostasis model assessment for insulin resistance
*HR*_max_: maximum heart rate
*HR*_rest_: resting heart rate
*HR*_target_: target heart rate
RCT: randomized controlled trial
