# Supplementary material for: Efficiency of an mHealth App and Chest-Wearable Remote Exercise Monitoring Intervention in Patients With Type 2 Diabetes: A Prospective, Multicenter Randomized Controlled Trial
Source: JMIR Mhealth Uhealth. 2021 Feb 9;9(2):e23338. doi: 10.2196/23338 (PMC7902189; doi:10.2196/23338)

Figure S1. Interface of exercise prescription on the app


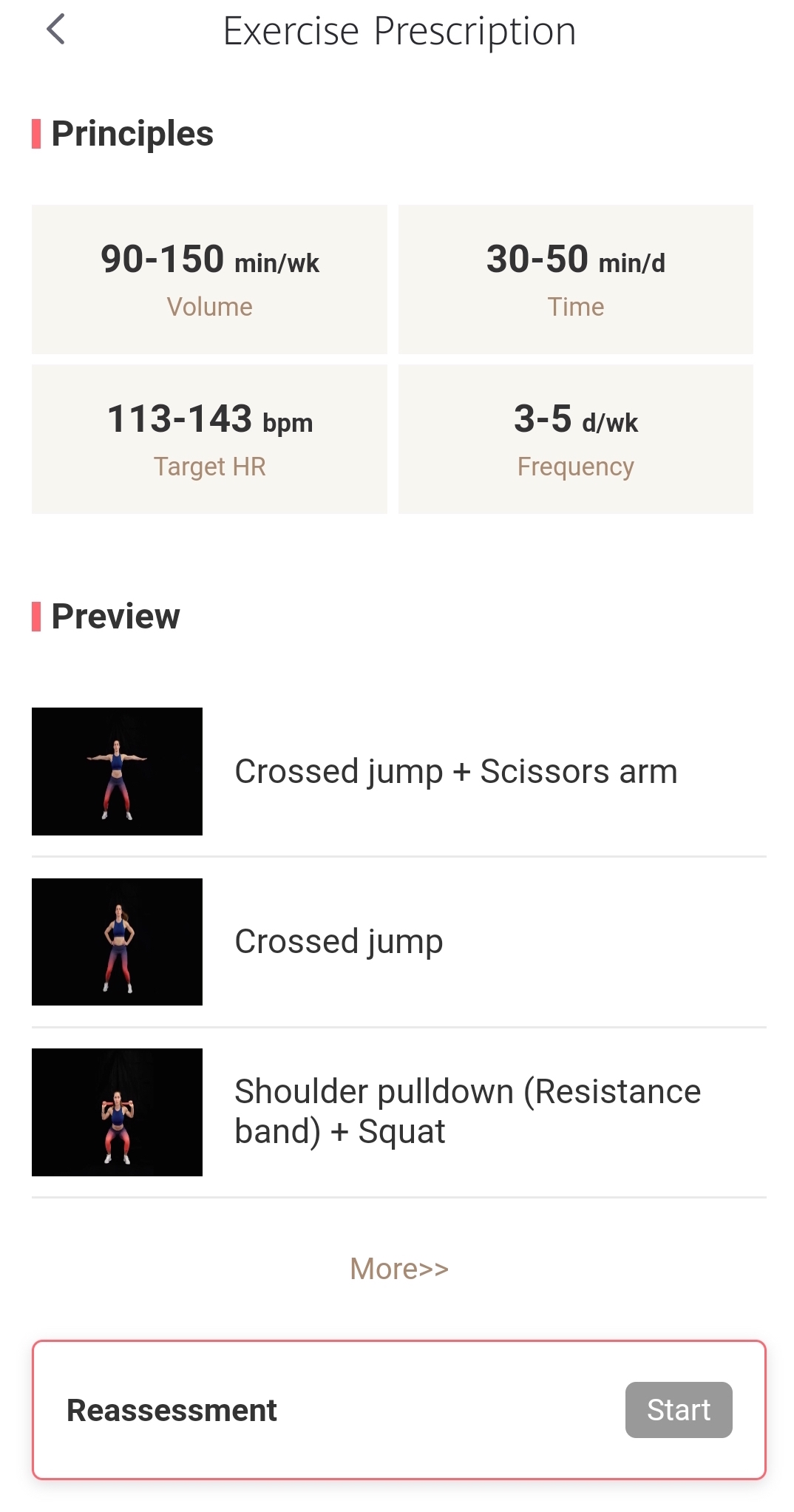


Figure S2. Interface of exercise preparation on the app


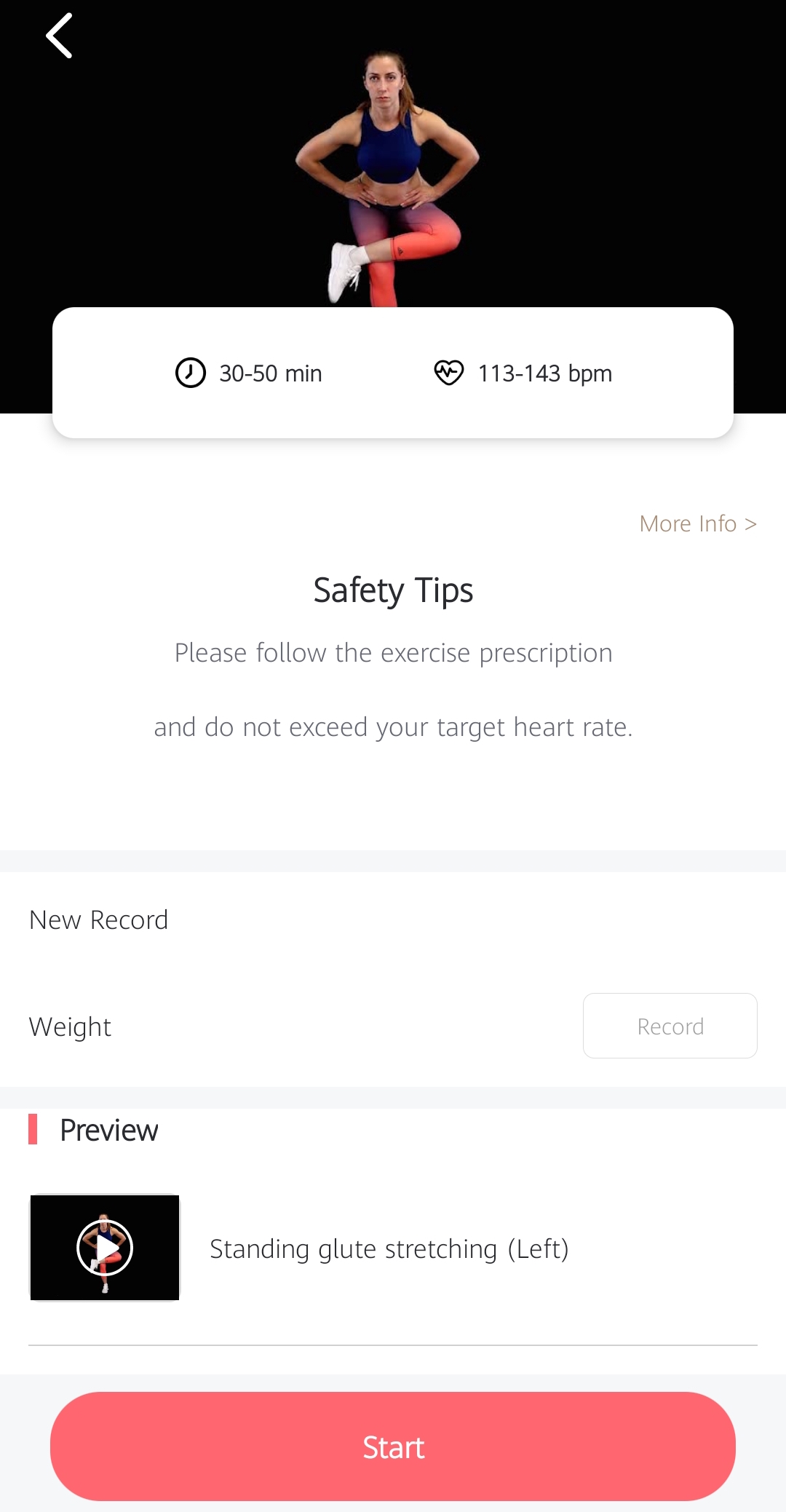


Figure S3. Interface of not reaching target heart rate during exercise


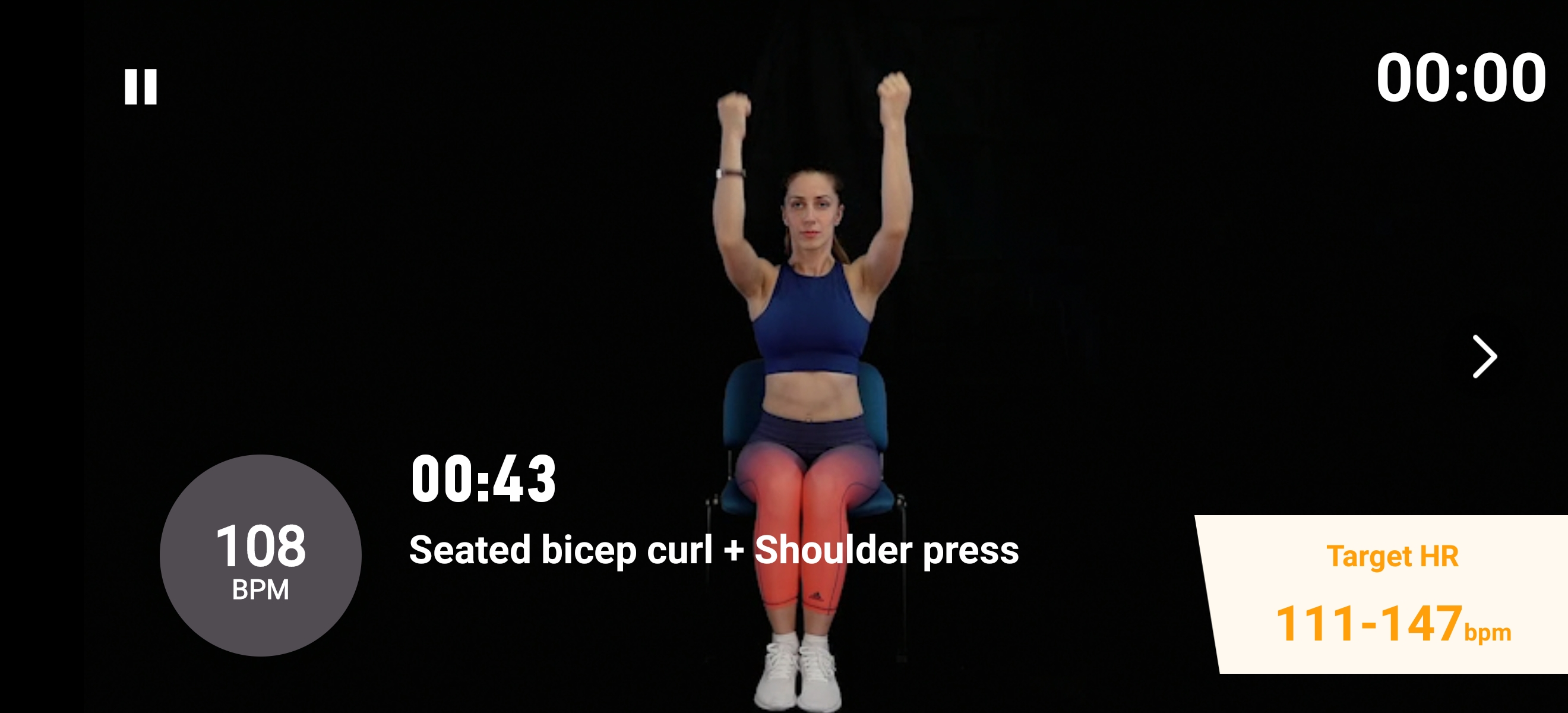


Figure S4. Interface of exercise record on the app


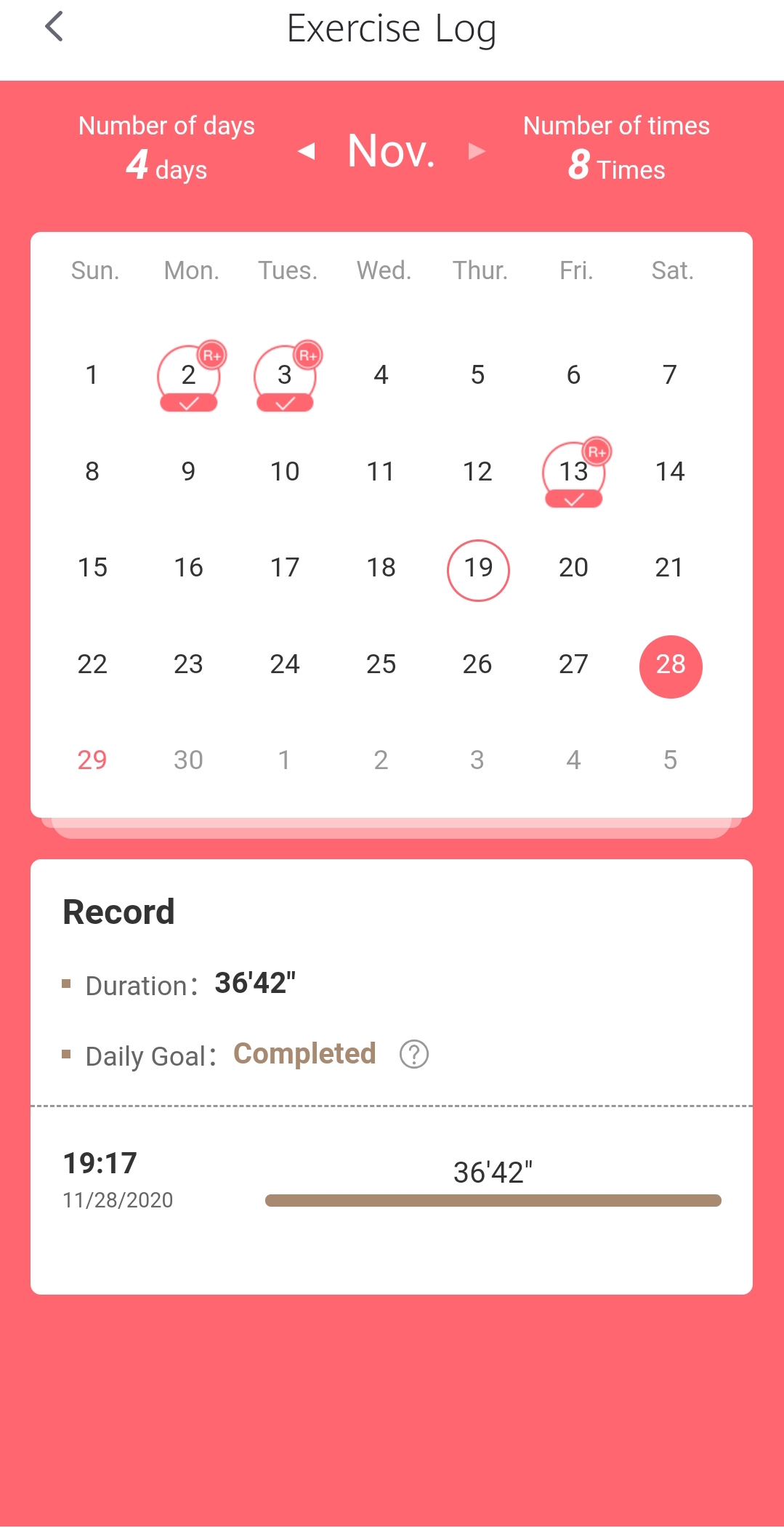

Supplement: Multimedia Appendix 1 [file mhealth_v9i2e23338_app1.docx]
